# Human cytomegalovirus seropositivity and its influence on oral rotavirus vaccine immunogenicity: a specific concern for HIV-exposed-uninfected infants

**DOI:** 10.1093/cei/uxae029

**Published:** 2024-03-28

**Authors:** Natasha Laban, Samuel Bosomprah, Roma Chilengi, Michelo Simuyandi, Caroline Chisenga, Harriet Ng’ombe, Kalo Musukuma-Chifulo, Martin Goodier

**Affiliations:** Enteric Disease and Vaccine Research Unit, Centre for Infectious Disease Research in Zambia, Lusaka, Zambia; Department of Infection Biology, Faculty of Infectious and Tropical Diseases, London School of Hygiene and Tropical Medicine, London, UK; Enteric Disease and Vaccine Research Unit, Centre for Infectious Disease Research in Zambia, Lusaka, Zambia; Department of Biostatistics, School of Public Health, University of Ghana, Accra, Ghana; Enteric Disease and Vaccine Research Unit, Centre for Infectious Disease Research in Zambia, Lusaka, Zambia; Enteric Disease and Vaccine Research Unit, Centre for Infectious Disease Research in Zambia, Lusaka, Zambia; Enteric Disease and Vaccine Research Unit, Centre for Infectious Disease Research in Zambia, Lusaka, Zambia; Enteric Disease and Vaccine Research Unit, Centre for Infectious Disease Research in Zambia, Lusaka, Zambia; Enteric Disease and Vaccine Research Unit, Centre for Infectious Disease Research in Zambia, Lusaka, Zambia; Department of Infection Biology, Faculty of Infectious and Tropical Diseases, London School of Hygiene and Tropical Medicine, London, UK; Flow Cytometry and Immunology Facility, Medical Research Council Unit, The Gambia at London School of Hygiene and Tropical Medicine, Fajara, Banjul, The Gambia

**Keywords:** rotavirus, vaccine, antibody, human cytomegalovirus, Zambia

## Abstract

Oral rotavirus vaccines demonstrate diminished immunogenicity in low-income settings where human cytomegalovirus infection is acquired early in childhood and modulates immunity. We hypothesized that human cytomegalovirus infection around the time of vaccination may influence immunogenicity. We measured plasma human cytomegalovirus-specific immunoglobulin M antibodies in rotavirus vaccinated infants from 6 weeks to 12 months old and compared rotavirus immunoglobulin A antibody titers between human cytomegalovirus seropositive and seronegative infants. There was no evidence of an association between human cytomegalovirus serostatus at 9 months and rotavirus-specific antibody titers at 12 months (geometric mean ratio 1.01, 95% CI: 0.70, 1.45; *P* = 0.976) or fold-increase in RV-IgA titer between 9 and 12 months (risk ratio 0.999, 95%CI: 0.66, 1.52; *P* = 0.995) overall. However, HIV-exposed-uninfected infants who were seropositive for human cytomegalovirus at 9 months old had a 63% reduction in rotavirus antibody geometric mean titers at 12 months compared to HIV-exposed-uninfected infants who were seronegative for human cytomegalovirus (geometric mean ratio 0.37, 95% CI: 0.17, 0.77; *P* = 0.008). While the broader implications of human cytomegalovirus infections on oral rotavirus vaccine response might be limited in the general infant population, the potential impact in the HIV-exposed-uninfected infants cannot be overlooked. This study highlights the complexity of immunological responses and the need for targeted interventions to ensure oral rotavirus vaccine efficacy, especially in vulnerable subpopulations.

## Teaser

Among rotavirus vaccinated infants, there was no evidence of an association between HCMV-IgM serostatus at 9 months with RV-IgA titer at 12 months (GMR 1.01, 95%CI: 0.70, 1.45; *P*  = 0.976). However, HIV-exposed-uninfected infants who were HCMV-IgM seropositive at 9 months old had a 63% reduction in RV-IgA geometric mean titers at 12 months compared to HIV-exposed-uninfected infants who were HCMV-IgM seronegative (geometric mean ratio 0.37, 95%CI: 0.17, 0.77; *P*  = 0.008). Created with BioRender.com

## Graphical Abstract

**Graphical Abstract:**
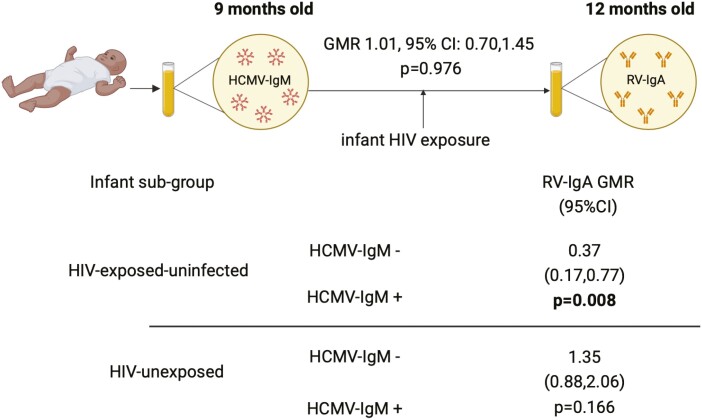

## Introduction

Rotavirus, a leading cause of diarrheal disease in children [1], remains a public health concern particularly in low- and middle-income countries (LMICs). The use of oral rotavirus vaccines (ORV) [2] has decreased the degree of diarrheal disease caused by rotavirus in children residing in LMICs especially in Africa [3, 4]. The impact has been to bring down hospitalizations for rotavirus diarrhea in those children aged 5 years and below [4]. However, these vaccines demonstrate diminished seroconversion rates in LMICs, a phenomenon not yet fully understood [5]. Zambia has seen a decrease in rotavirus diarrhea since ORV introduction [6, 7], but low seroresponse rates persist, estimated between 27% and 60% [8, 9]. Researchers have pinpointed numerous factors that could play a role [5], but the impact of persistent viral infections when receiving the vaccine is yet to be examined.

Human cytomegalovirus (HCMV), a β-herpesvirus [10], is common and can be transmitted congenitally and during nursing across different regions including Africa [11]. HCMV infection occurs early in childhood in Africa, with over 80% of infants infected by their first birthday [12]. In Zambia, about 83% of infants acquire HCMV infection by 18 months of age [13]. The high HCMV prevalence, its effects on host immunity and the observed poor ORV immunogenicity in these settings necessitate longitudinal studies to investigate temporal associations with childhood vaccine responses as argued by others [14].

Studies regarding HCMV’s influence on immunogenicity of childhood vaccines in Africa are scarce and show inconsistent findings. For some vaccines such as measles, HCMV has been found to have no effect [15], beneficial effects [16, 17] but also associated with reduced immune responses [17]. The effect of HCMV on other vaccinations such as meningococcal [16], Hepatitis B (HepB) [15, 18], diphtheria-pertussis-tetanus (DPT) [15, 17–19], and Bacille Calmette-Guérin (BCG) [15, 19] has been conflicting, with studies showing varying associations with vaccine-induced cellular and humoral responses. In Zambia, no significant associations between HCMV and oral polio vaccine antibody responses have been observed [20].

The current ambiguity in the direction of HCMV’s influence on infant vaccine responses and the absence of data for ORV signal the need for additional research. This study explores HCMV-IgM seroconversion in the first year of life in Zambia and its effect on rotavirus specific antibody responses among rotavirus vaccinated infants. It addresses the complex relationship between HCMV and vaccine immunogenicity in the context of low-income settings, infant health, and current vaccination strategies, shedding light on an understudied yet vital area of pediatric infectious disease management.

## Materials and methods

### Study design and participants

We conducted a longitudinal study nested within an open label, two-arm parallel group, randomized controlled trial (RCT). The RCT compared a two-dose (control arm) and three-dose (intervention arm) Rotarix™ vaccination schedule among Zambian infants. The details of the study design have been published elsewhere [21]. Briefly, 214 infants aged 6–12 weeks were enrolled in the parent RCT and followed up until they were 3 years of age between 2018 and 2021. During the first year of follow-up, all infants were given two doses of an ORV (Rotarix, GlaxoSmithKline) with the first dose administered from 6 weeks old and the second dose administered from 10 weeks old, along with polio, BCG, DPT-HepB-Hib and pneumococcal conjugate vaccines as part of the regular Zambia national immunization schedule. When the infants reached 9 months of age, they were randomly assigned to either a control arm (receiving only a measles-rubella (MR) vaccination) or an intervention arm (receiving MR vaccine and a third dose of Rotarix).

**Figure 1.**
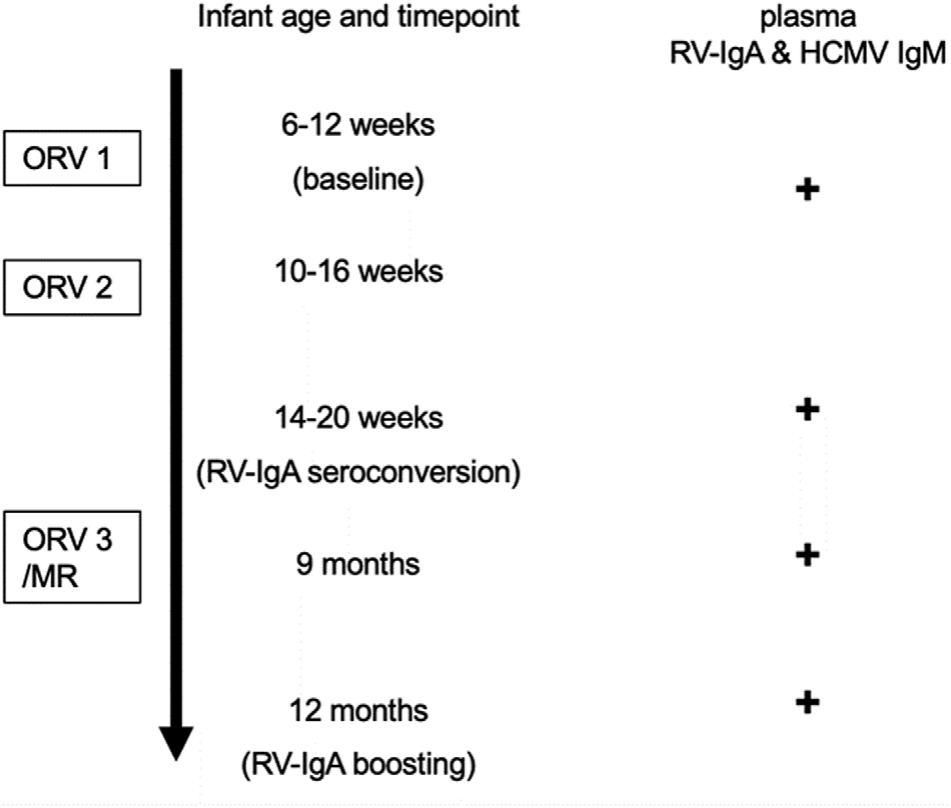
Study design. An illustration of the parent RCT study design and plasma collection timepoints.

Plasma samples were collected at specific intervals: at enrollment (baseline, aged 6-12 weeks) before the first Rotarix dose, 1 month after the second Rotarix dose (aged 14-20 weeks, when vaccine seroconversion was determined), at 9 months (before receipt of the third Rotarix dose and/or MR vaccine), and at 12 months (when the immune-boosting effect of third Rotarix dose was assessed; Fig. 1). These plasma samples were tested for rotavirus-specific immunoglobulin A (RV-IgA) antibodies. RV-IgA seropositivity was defined as an RV-IgA titer ≥ 20 units/ml. Vaccine seroconversion was defined as a 4-fold or greater change in RV-IgA antibody titer 1 month after dose two of Rotarix if pre-vaccination titer was less than 20 U/ml [21].

For this nested study, infants with available RV-IgA results and sufficient plasma sample to test for HCMV-IgM antibodies at baseline and at least one of the three subsequent time points up to 12 months of age were included. The design thus facilitated the examination of the association between HCMV infection and ORV immunogenicity by focusing on multiple factors and time points, enabling a comprehensive analysis of the relationship.

### Laboratory procedures

#### Determination of HCMV serostatus:

HCMV-IgM antibodies were measured using enzyme-linked immunosorbent assay (ELISA) kits from Demeditec Diagnostics GmbH (Germany) and Alpha Diagnostic International (USA). The procedure followed the manufacturer’s guidelines. Infant plasma samples were diluted and added to a 96-well microtiter plate that had been precoated with a purified HCMV antigen. To detect any HCMV-IgM in these samples, they were treated with horse-radish peroxidase (HRP)-conjugated anti-human IgM. Subsequently, the tetramethylbenzidine (TMB) substrate was added, initiating an enzyme-substrate hydrolysis reaction, which resulted in color development. The color’s absorbance was immediately measured at a 450-nm wavelength using an Epoch 2 microplate reader by Agilent (South Africa). Proper quality control of each experiment was ensured using the calibrators and controls provided in the kit. To check consistency in results, selected HCMV seropositive samples were tested with both kits. The test outcomes for HCMV-IgM serostatus in the plasma samples were categorized as either positive or negative based on specific cut-off control values for each experiment.

#### Quantification of rotavirus-specific immunoglobulin A:

 The measurement of rotavirus-specific immunoglobulin A (RV-IgA) was carried out using a sandwich ELISA method, as outlined in the parent rotavirus vaccine trial [21]. In the procedure, infant plasma samples were placed on a 96-well microtitre plate, which was coated with alternating columns of rotavirus infected and uninfected cell lysate. To detect the RV-IgA, the samples underwent a subsequent treatment with biotinylated anti-human IgA and an avidin-biotin-peroxidase complex. The addition of the o-phenylenediamine dihydrochloride substrate initiated a color change, whose intensity was measured at a 492-nm wavelength with a microplate reader. The concentration of RV-IgA was determined based on these readings, compared against a standard curve created from a known rotavirus IgA plasma standard.

### Statistical analysis

Background characteristics were summarized with mean and standard deviation (SD) or median and interquartile range (IQR) for continuous variables. Categorical variables were summarized using frequency and proportion. Pearson’s chi-square or Fisher’s exact test was used to compare the distribution of categorical background characteristics by HCMV-IgM serostatus at 9 months and 4-fold change in RV-IgA titers between 9 and 12 months. For RV-IgA titers at 12 months, we used Student *t*-test on log-transformed values. For anthropometric indices, we calculated *z*-scores using the 2006 World Health Organization child growth standards. The exposure of interest was HCMV-IgM serostatus at 9 months and the primary outcome was RV-IgA titers at 12 months. Secondary outcome was proportion with 4-fold-change in RV-IgA titers between 9 and 12 months. The primary analysis was conducted among infants that had HCMV serostatus result at the 9-month time point and RV-IgA titer result at both the 9- and 12-month timepoints.

We used linear regression model of log-transformed RV-IgA titers to estimate the effect of HCMV-IgM serostatus at 9 months or cumulative HCMV-IgM seroconversion by 9 months on RV-IgA GMT at 12 months, adjusting for potential confounders. *P*-value of <0.05 was considered statistically significant. The RV-IgA titer below the range of the standard curve were imputed as “1” prior to log-transformation. We used generalized linear model, adjusted for potential confounders, to estimate the effect of HCMV-IgM serostatus at 9 months or cumulative HCMV-IgM seroconversion by 9 months on the proportion with a 4-fold or greater change in RV-IgA titers between 9 and 12 months. In a subgroup analysis, we used likelihood ratio test of interaction to investigate whether the effect of HCMV-IgM serostatus at 9 months on RV-IgA titer at 12 months varied by two-dose versus three-dose vaccination or by infant human immunodeficiency virus (HIV) exposure. In exploratory analyses, we examined the proportion of infants testing seropositive for HCMV-IgM for each time point and the relationship between HCMV-IgM point seropositivity or cumulative HCMV-IgM seroconversion and vaccine seroconversion after two dose vaccination. All analyses were performed in Stata 17 (StataCorp, College Station, TX, USA) and GraphPad Prism v9 (GraphPad Software, LLC).

## Results

**Figure 2.**
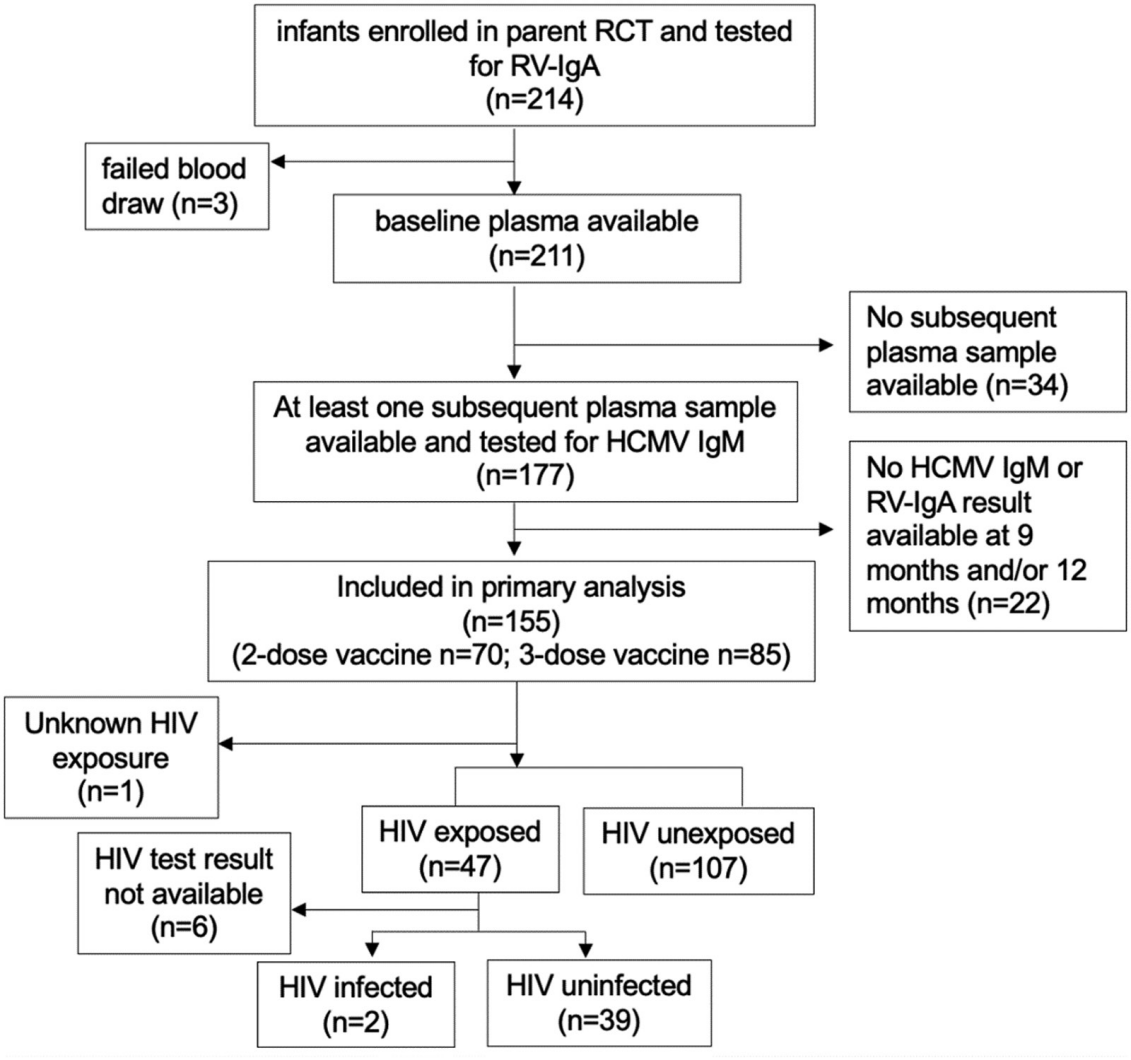
Flow diagram of infant samples included in the study. A schema of the participant flow and criteria used in the selection of plasma samples and infant subgroups included in analysis.

The parent Rotarix RCT enrolled and quantified RV-IgA titers for 214 infants of which 177 had sufficient plasma available at baseline and for at least one other timepoint; these were also tested for HCMV-IgM. Of these a total of 155/177 (88%) infants met the criteria for inclusion in our primary analysis and included HIV-exposed-infected, HIV-exposed-uninfected and HIV unexposed infants (Fig. 2).

### Infant baseline characteristics

As shown in Table 1, among the infants included in the primary analysis (*n* = 155), median age at baseline was 6 weeks, majority were vaginally delivered (95%) at full term (94%), with normal birthweight (90%), and were predominantly breastfed exclusively (94%). Most infants came from homes with less than ideal water and sanitation, often sharing toilets with other households (79%) and getting water from public sources (65%) and approximately one-third of infants had HIV-positive mothers. There were 60 HCMV-IgM seropositive and 95 HCMV-IgM seronegative infants at 9 months of age. There was no statistically significant relationship between baseline characteristics and HCMV-IgM serostatus at 9 months of age (Table 1).

**Table 1.** Baseline characteristics by HCMV-IgM serostatus at 9 months

	HCMV-IgM serostatus at 9 months old			
		Seronegative	Seropositive	
Characteristic	*n* (% of total)	*n* (% of total)	*n* (% of total)	*P* value
Age				
Median(IQR)	6 (6.6)	6 (6.6)	6 (6.6)	0.927
Sex				
Female	73 (47.1)	41 (43.2)	32 (53.3)	0.249
Male	82 (52.9)	54 (56.8)	28 (46.7)	
Gestation				
Full-term	146 (94.2)	89 (93.7)	57 (95.0)	1.000
Pre-term	9 (5.8)	6 (6.3)	3 (5.0)	
Mode of delivery				
Caesarean	8 (5.2)	4 (4.2)	4 (6.7)	0.712
Vaginal	147 (94.8)	91 (95.8)	56 (93.3)	
Feeding				
Breastmilk	145 (93.5)	91 (95.8)	54 (90.0)	0.187
Breastmilk + formula	10 (6.5)	4 (4.2)	6 (10.0)	
Birth weight, kg (*n* = 154)				
< 2.5	16 (10.4)	10 (10.5)	6 (10.2)	1.000
≥ 2.5	138 (89.6)	85 (89.5)	53 (89.8)	
Stunting (LAZ < −2)				
No	129 (83.2)	75 (79.0)	54 (90.0)	0.081
Yes	26 (16.8)	20 (21)	6 (10.0)	
Malnourished (WLZ < -2)				
No	152 (98.1)	92 (96.8)	60 (100.0)	0.284
Yes	3 (1.9)	3 (3.2)	0 (0.0)	
Maternal HIV (*n* = 154)				
Negative	107 (69.5)	65 (68.4)	42 (71.2)	0.857
Positive	47 (30.5)	30 (31.6)	17 (28.8)	
Toilet facility sharing across households				
Not shared	32 (20.6)	22 (23.2.7)	10 (16.7)	0.416
Shared	123 (79.4)	73 (76.8)	50 (83.3)	
Water source				
Piped into household	55 (35.5)	35 (36.8)	20 (33.3)	0.731
Wells/public taps and boreholes	100 (64.5)	60 (63.2)	40 (66.7)	
Number of children in household				
1–3	122 (78.7)	76 (80.0)	46 (76.7)	0.872
4–6	29 (18.7)	17 (17.9)	12 (20.0)	
7–9	4 (2.6)	2 (2.1)	2 (3.3)	
Total	155 (100)	95 (61.3)	60 (38.7)	

We additionally assessed for associations between infant baseline characteristics and the primary outcomes of RV-IgA at 12 months old and secondary outcome of 4-fold increase in RV-IgA titer between 9 and 12 months old. We found that infants residing in households that did not share toilet facility had higher RV-IgA GMT at 12 months compared to infants from households with shared toilet facility (*P* = 0.027) but for all other baseline characteristics, no statistically significant relationship was observed (Supplementary Table S1). There was no statistically significant relationship observed between infant baseline characteristics and 4-fold or greater increase in RV-IgA titer between 9 and 12 months (Supplementary Table S2).

### HCMV-IgM serostatus by age

**Figure 3.**
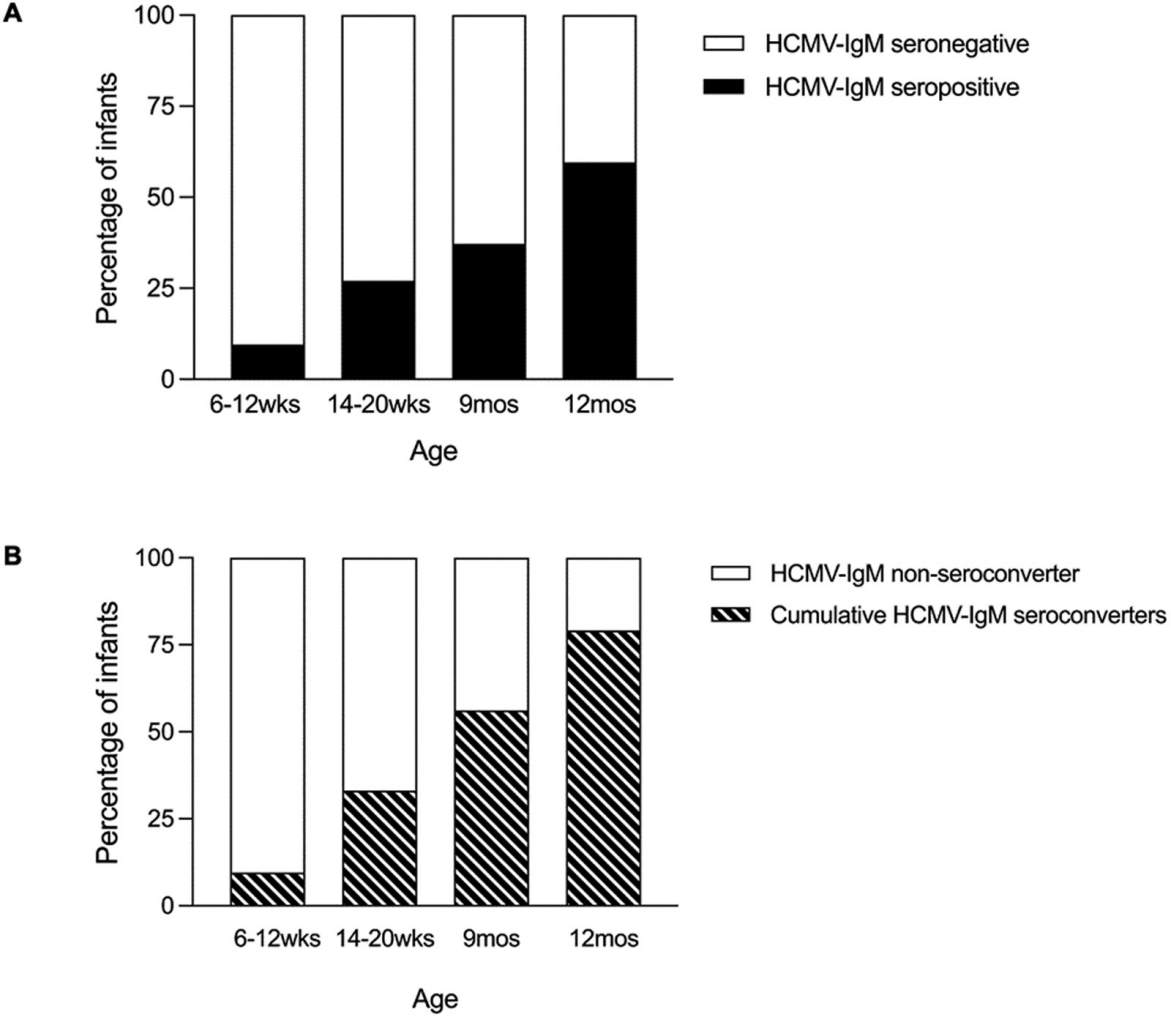
Infant HCMV-IgM serostatus by age. The percentage of HCMV-IgM seropositive and seronegative infants (panel A) and cumulative HCMV-IgM seroconverters and non-seroconverters (panel B) at each age timepoint is shown as bars (*n* = 148).

To assess HCMV-IgM seropositivity by age, we included infants that had an HCMV-IgM result at all the four age timepoints 6-12 weeks, 14-20 weeks, 9 months, and 12 months (*n* = 148) out of the 177 that had a baseline and at least one follow-up sample collected. The proportion of infants that were HCMV-IgM seropositive at each age timepoint increased from 9.5% (14/148) at ages 6-12 weeks, to 27.0% (40/148) at 14-20 weeks, 37.2% (55/148) at 9 months and 59.5% (88/148) at 12 months (Fig. 3A). We also assessed cumulative HCMV-IgM seroconversion with infants defined as HCMV-IgM seroconverters when they became HCMV-IgM seropositive after having HCMV-IgM seronegative results for all preceding timepoints. By 12 months old, the cumulative HCMV-IgM seroconversion was 79.1% (117/148), and 20.9% (31/148) infants were HCMV-IgM seronegative throughout (Fig. 3B).

### Effect of HCMV-IgM serostatus on rotavirus antibody response

In the overall study population (*n* = 155), the RV-IgA GMT were 1.3 units/ml (95%CI: 1.1, 1.6) at 6–12 weeks (*n* = 154), 3.2 units/ml (95%CI: 2.3, 4.4) at 14–20 weeks (*n* = 149), 6.8 units/ml (95%CI: 4.7, 9.8) at 9 months (*n* = 155) and 24.8 units/ml (95%CI:16.6, 36.9) at 12 months (*n* = 155). There were 7/154 (4.6%), 27/149 (18%), 49/155 (31.6%), and 84/155 (54.2%) infants that were RV-IgA seropositive at 6–12 weeks, 14–20 weeks, 9 months, and 12 months, respectively. A total of 148 infants had RV-IgA results at both 6–12 and 14–20 weeks and among these, 40/148 (27.0%) were vaccine seroconverters and 108/148 (73%) were vaccine non-seroconverters.

At 12 months, the RV-IgA geometric mean titers (GMT) was 23.2 units/ml (95%CI: 12.32, 43.5) among infants seropositive for HCMV-IgM at 9 months and 25.8 units/ml (95%CI: 15.3, 43.7) among those that were HCMV-IgM seronegative. As shown in Table 2, irrespective of the number of vaccine doses and after adjusting for the potential confounding effect of sex, breastfeeding, stunting, wasting, and toilet facility, there was no statistically significant difference in RV-IgA GMT at 12 months between HCMV-IgM seropositive and HCMV-IgM seronegative infants at 9 months (geometric mean ratio (GMR) 1.01, 95%CI: 0.70,1.45; *P* = 0.976). A 4-fold or greater increase in RV-IgA titer between 9 and 12 months of age was observed in 61/155 infants (39.4%) and 23/60 (37.3%) were from HCMV-IgM seropositive and 38/95 (40.0%) were from HCMV-IgM seronegative infants. Irrespective of the number of vaccine doses and after adjusting for the potential confounding effect of sex, breastfeeding, stunting, wasting, and toilet facility, there was no evidence of an association between HCMV-IgM serostatus at 9 months and the 4-fold or greater increase in RV-IgA titer (risk ratio (RR) 0.99, 95% CI: 0.66,1.52; *P* = 0.995) (Table 2). Similarly, we found no statistically significant relationship between cumulative HCMV-IgM seroconversion status by 9 months and RV-IgA GMT at 12 months (GMR 1.24, 95%CI: 0.86, 1.78; *P* = 0.239) or a 4-fold or greater increase in RV-IgA titer between 9 and 12 months (RR 0.88, 95% CI: 0.59,1.32; *P* = 0.539) (Table 2).

**Table 2.** Effect of HCMV-IgM serostatus on RV-IgA titers and 4-fold increase

HCMV at 9 months	Number infants *N* (% of total)	RV-IgA GMT at 12 months (95%CI)	RV-IgA *GMR at 12 months (95% CI)	*P*-value	Mounted ≥ 4-fold rise in RV-IgA titer between 9 and 12 months *n* (%)	*RR (95% CI)	*P*-value
HCMV-IgM point serostatus							
HCMV IgM -	95 (61.3)	25.8 (15.3,43.7)	1	0.976	38 (40.0)	1	0.995
HCMV IgM +	60 (38.7)	23.2 (12.3,43.5)	1.01 (0.70,1.45)		23 (38.3)	0.99 (0.66,1.52)	
HCMV-IgM cumulative seroconversion							
HCMV-IgM ns	67 (43.2)	20.67 (11.7,36.6)	1	0.239	28 (41.8)	1	0.539
HCMV-IgM s	88 (56.8)	28.4 (16.2,49.8)	1.24 (0.86,1.78)		33 (37.5)	0.88 (0.59,1.32)	
Total	155 (100)	24.76 (16.6,36.9)	—		61(39.4)		—

Abbreviations: CI (confidence interval), ns (non-seroconverter), RR (risk ratio), s (seroconverter).

^*^Estimates were adjusted for sex, breastfeeding, stunting, wasting, and toilet facility.

### Subgroup analysis by infant HIV exposure and vaccine dose schedule

**Figure 4.**
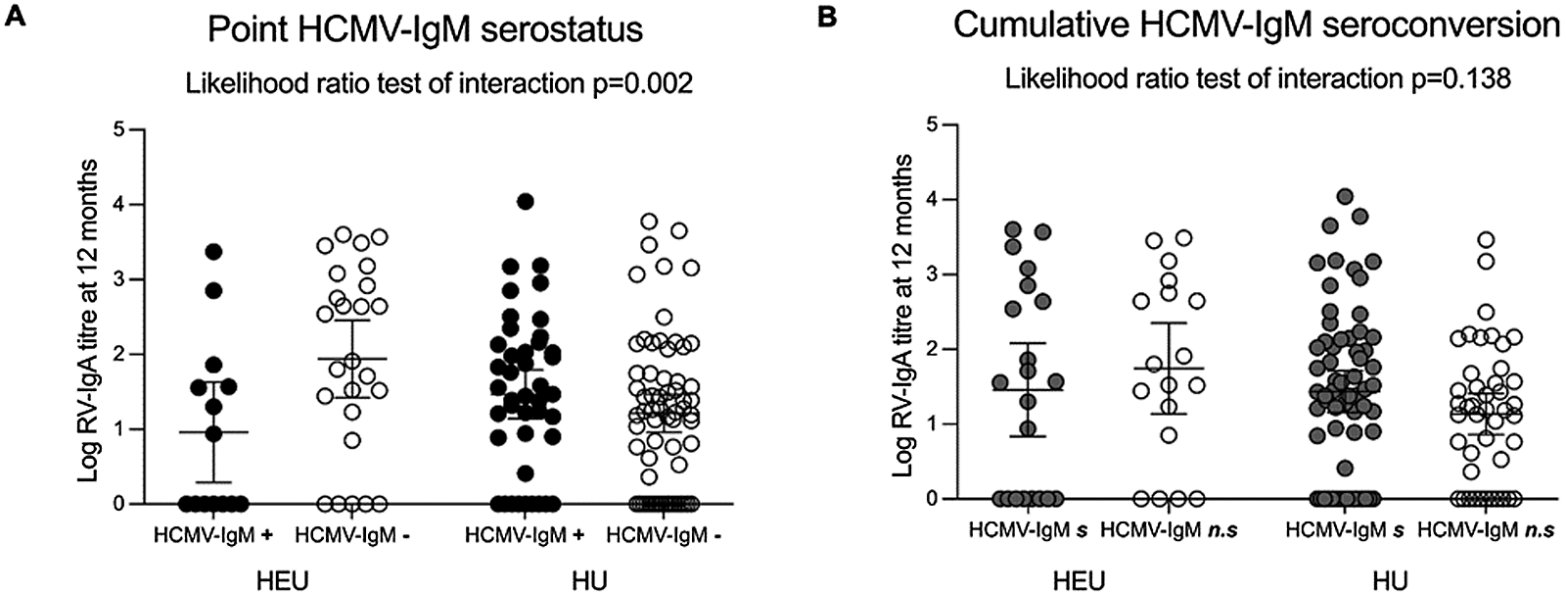
RV-IgA titres at 12 months infant age by point and cumulative HCMV-IgM serostatus at 9 months old stratified by infant HIV exposure. Each circle represents the log-transformed RV-IgA titer for a single infant (*n* = 146) among HIV-exposed-uninfected (HEU, *n* = 39) and HIV-unexposed (HU, *n* = 107) infants. Black and white circles indicate HCMV-IgM seropositive (+) and HCMV-IgM seronegative (−) infants at 9 months, respectively (panel A). Grey and white circle indicate cumulative HCMV-IgM seroconverting (*s*) and non-seroconverting (*n.s.*) infants at 9 months respectively (panel B). Solid horizontal bar and error bars indicates the mean value with 95% confidence intervals.

Of the 47/154 infants maternally exposed to HIV, 41/47 (87.2%) had an HIV result available. Of these, 39/41 (95.1%) were uninfected (HIV-exposed-uninfected, HEU), and 2/41 (4.9%) were infected (HIV-exposed-infected). We excluded the HIV-exposed-infected (*n* = 2) from subsequent analysis. As shown in Fig. 4A, analysis of HEU and HIV-unexposed infants (HU, *n* = 146), demonstrated an effect of point HCMV-IgM serostatus at 9 months on RV-IgA titers at 12 months according to infant HIV exposure status (likelihood ratio test of interaction *P* = 0.002). In contrast, there was no evidence of an interaction between infant HIV status and the effect of cumulative HCMV-IgM seroconversion by 9 months on RV-IgA titers at 12 months (likelihood ratio test of interaction *P* = 0.138) in this grouping (Fig. 4B).

**Figure 5.**
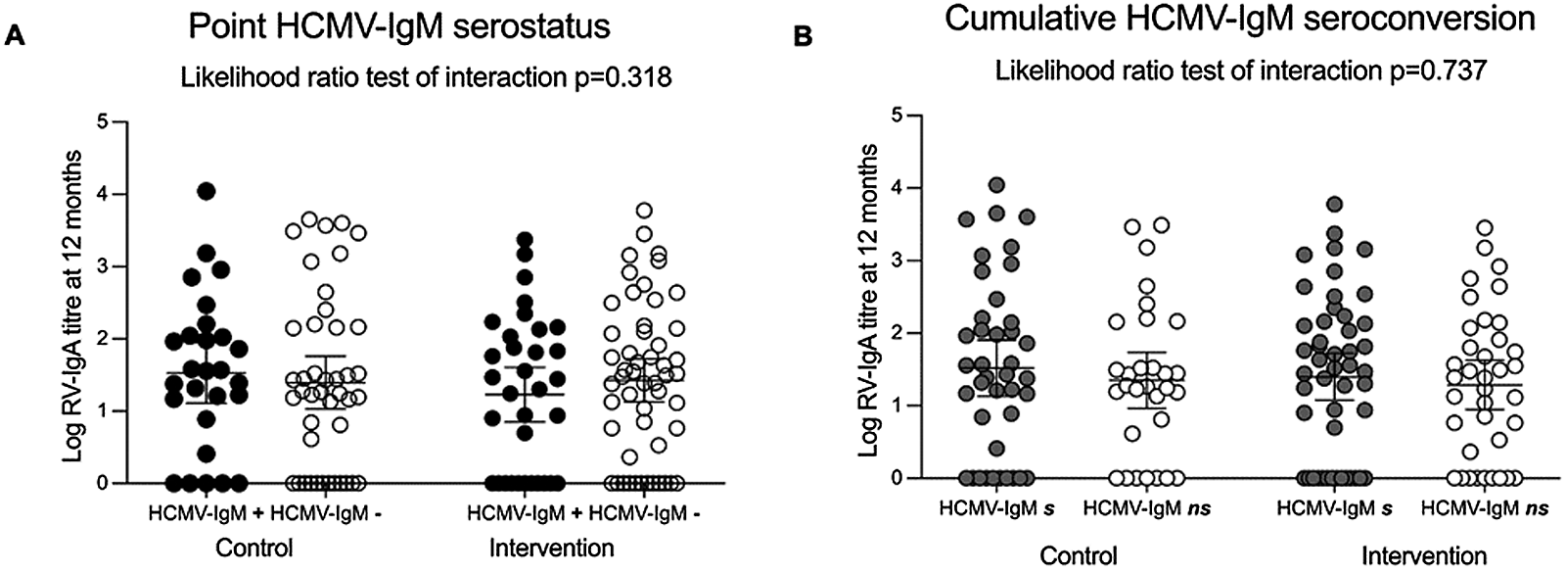
Mean rotavirus antibody titers at 12 months infant age by point and cumulative HCMV-IgM serostatus at 9 months old stratified by vaccine dose schedule. Each circle represents the log-transformed RV-IgA titer for a single infant (*n* = 155) randomized to the intervention arm (*n* = 85) and control arm (*n* = 70). Black and white circle indicates HCMV-IgM seropositive (+) and seronegative (−) infants at 9 months, respectively (panel A). Grey and white circle indicates cumulative HCMV-IgM seroconverting (*s*) and non-seroconverting (*ns*) infants at 9 months, respectively (panel B). Solid horizontal bar and error bars indicates the mean value with 95% confidence intervals.

As shown in Fig. 5, we found no evidence that the effect of HCMV-IgM seropositivity at the 9 months timepoint (Fig. 5A) or cumulative HCMV-IgM seroconversion by 9 months (Fig. 5B) on RV-IgA titer at 12 months of age varied by the vaccine dose schedule for the entire cohort (two versus three doses of Rotarix, *n* = 155) (likelihood ratio test of interaction *P* = 0.318 and *P* = 0.737, respectively).

Among the HU infants (*n* = 107), there was no statistically significant difference in RV-IgA GMT at 12 months between infants that were HCMV-IgM seropositive (GMT 29.4, 95%CI: 13.9, 61.9) and HCMV-IgM seronegative (GMT 16.4, 95%CI: 9.1, 29.4) at 9 months (GMR 1.35, 95%CI: 0.88, 2.06; *P* = 0.166) (Table 3). In the HEU group, there was evidence that the RV-IgA GMT at 12 months was decreased by 63% in infants that were HCMV-IgM seropositive compared to those that were HCMV-IgM seronegative at 9 months (GMR 0.37, 95%CI: 0.17,0.77; *P* = 0.008) (Table 3).

**Table 3.** Effect of HCMV-IgM serostatus on RV-IgA titers by infant HIV status

Subgroups^a^)	Number of infants *N* (% of total)	RV-IgA GMT at 12 months (95%CI)	RV-IgA *GMR (95% CI)	*P*-value
HIV-unexposed	107 (73.3)	20.6 (13.0,32.5)	1.35 (0.88,2.06)	0.166
HCMV-IgM-	65 (60.8)	16.4 (9.1, 29.4)		
HCMV-IgM+	42 (39.2)	29.4 (13.9, 61.9)		
HIV-exposed-uninfected	39 (26.7)	38.8 (14.8, 102.0)	0.37 (0.17,0.77)	**0.008**
HCMV-IgM-	25 (64.1)	87.2 (26.5, 286.4)		
HCMV-IgM+	14 (35.9)	9.1 (2.0, 42.8)		
Total	146	24.4 (16.0, 37.1)		

^a^Likelihood ratio test of interaction *P*-value = 0.002. *Estimates were adjusted for sex, breastfeeding, stunting, waisting, and toilet facility.

**Figure 6.**
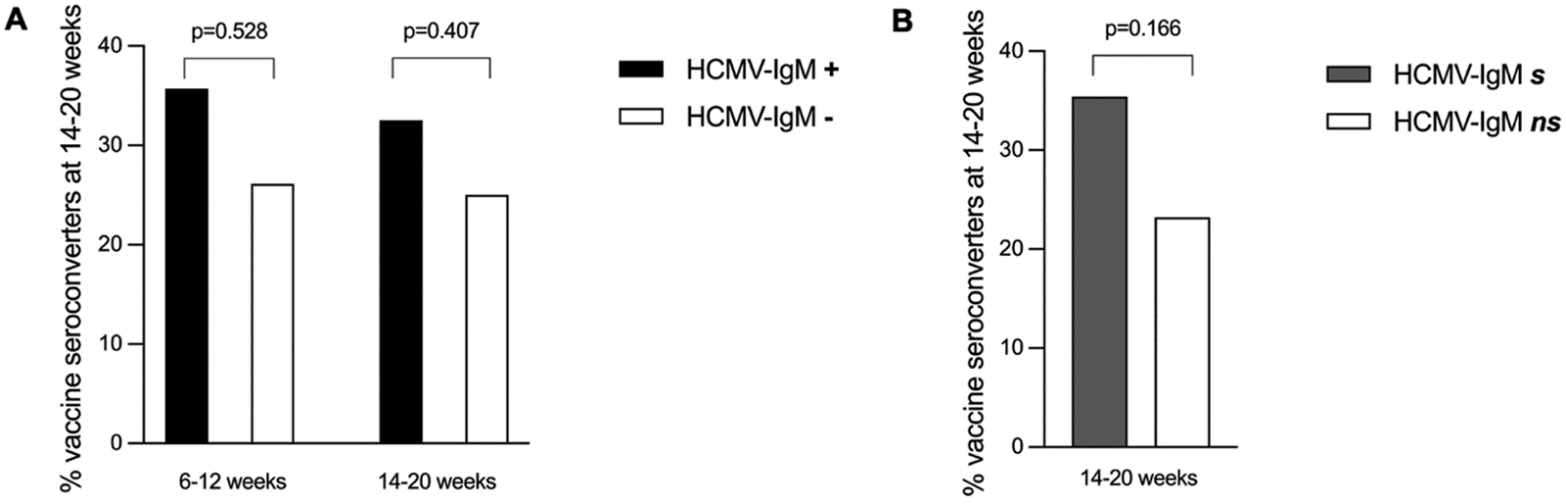
Vaccine seroconversion by HCMV-IgM serostatus. Each bar represents the percent vaccine seroconverters at 14–20 weeks (1 month after two dose vaccination) among infants that were HCMV-IgM seropositive (*n* = 14) and HCMV IgM seronegative (*n* = 134) before vaccination at 6–12 weeks old (*n* = 148); HCMV IgM seropositive (*n* = 40) and HCMV IgM seronegative (*n* = 108) at 14–20 weeks old (*n* = 148); and cumulative HCMV-IgM seroconverting (*n* = 48) and non-seroconverting (*n* = 99) infants at 14–20 weeks old (*n* = 147). Black and white bars indicate HCMV-IgM seropositive (+) and seronegative (−) infants, respectively (panel A). Grey and white bars indicate cumulative HCMV-IgM seroconverting (s) and non-seroconverting (ns) infants respectively (panel B).

As shown in Fig. 6, there was no statistically significant difference in the frequency of vaccine seroconversion one month after two dose vaccination either by HCMV-IgM serostatus at 6-12 weeks (*n* = 148, *P* = 0.528) and 14–20 weeks timepoints (*n* = 148, *P* = 0.407) or by cumulative HCMV-IgM seroconversion at 14–20 weeks timepoint (*n* = 147, *P* = 0.166).

## Discussion

Our study aimed to investigate the influence of HCMV infection around the time of oral rotavirus vaccination on the vaccine immunogenicity in a low-income setting where early childhood HCMV infection is prevalent and may modulate immune responses. We measured HCMV-IgM in vaccinated infants when they were 9 months of age, which coincided with the time of a third dose of ORV. We specifically examined any association between the presence of HCMV-IgM (indicative of recent HCMV infection or reactivation) and the antibody response to rotavirus vaccine (measured as RV-IgA titers). Overall, there was no evidence of association, at 5% level of significance, between the presence of HCMV-IgM at 9 months of age and RV-IgA titers at 12 months. This suggests that for the majority of infants, HCMV infection does not seem to notably affect the vaccine’s immunogenicity. However, among HEU infants who were HCMV-IgM seropositive at 9 months, a 63% reduction in RV-IgA titer at 12 months was observed compared to their HEU-HCMV-IgM seronegative counterparts. This points to a possible-specific immune modulation effect of HCMV in HEU infants in our setting.

Our findings are similar to what has been reported for another orally administered pediatric vaccine, oral polio vaccine (OPV), where a study in Zambia showed no effect of HCMV DNAemia or HCMV serostatus in 18-month-old infants on poliovirus antibody titers or proportion of infants with seroprotective levels in the overall study population [20]. In contrast to our findings on oral rotavirus vaccine, while significantly reduced poliovirus antibody responses were observed in maternally HIV exposed infants and HIV seropositive infants, the OPV study did not find any difference in poliovirus antibody responses by HCMV DNAemia or HCMV serostatus among the HEU infants [20]. Notably, trends of reduced OPV antibody responses among HIV-positive infants that had HCMV DNAemia compared to those without HCMV DNAemia were observed [20] although differences in determination of HCMV serostatus with our study (HCMV-IgG versus IgM) may limit comparisons. In our rotavirus vaccine study and the OPV study, while vaccine immunogenicity in the broader population was not impacted by HCMV infection, reduction of vaccine responses was seen within specific subgroups (HEU- and HIV-positive infants). This nuanced finding underscores the complexity of immunologic responses, especially in populations with various health challenges. Interestingly, in studies elsewhere with reported HIV prevalence of below 5%, HCMV infected infants are observed to have reduced antibody responses to tetanus toxoid after DPT vaccination and lowered T-cell effector responses post measles vaccination compared to HCMV uninfected infants despite no impact on infants’ attainment of vaccine-specific seroprotective levels; however, the infant HIV status in these studies was not ascertained [15, 17]. We could not speculate on the clinical significance of the reduced RV-IgA titer in HEU HCMV-IgM seropositive infants as there is currently no defined seroprotective threshold level of RV-IgA; however, higher RV-IgA titers are associated with reduced risk of rotavirus infection and diarrhea [22]. Thus, factors like HCMV associated with reductions in these RV-IgA responses among HEU infants have the potential to negatively impact overall vaccine efficacy and effectiveness.

HCMV affects the immune system by committing a substantial proportion of T cells toward its immune response [23] and causing accumulation of differentiated immune cells [23–25] and restricted T-cell repertoires [26] characteristic of immune senescence. HCMV can also disrupt antigen presentation to T cells, suppress immune effector function, and limit immune cell proliferation [27–29]. Expansion of differentiated T-cell immunity by HCMV is consistent with immunological profiles observed in HEU infants compared to HIV unexposed infants [30]. In the HEU population, HCMV may therefore attenuate vaccine immune responses via this T-cell immune perturbation [31]. We found evidence of an impact of HCMV on rotavirus-specific antibody response in HEU infants thus for rotavirus vaccines, further studies investigating the effect of HCMV infection on T-cell immune responses to vaccination in HEU infants are merited to elucidate such possible effects. HCMV also alters intestinal microbiome [32] favoring increased composition of *Bacteroidetes* [33]. Abundance of specific *Bacteroidetes* genera, *Bacteroides* and *Prevotella*, has been significantly correlated with a lack of seroresponse to rotavirus vaccination in infants [34] and is reported to be significantly increased in HEU compared to HIV unexposed infants [35]. Human microbiome colonization and immune development are intimately related and influence infant immune responses to vaccines [36]. Early life HCMV may therefore be an important determinant of oral vaccine responses in HEU via its effect on the intestinal microbiome composition but additional studies are needed to confirm this.

A major strength of this study is its focus on a vulnerable population (infants) within a low-income setting. We addressed an important prevailing question as to why oral rotavirus vaccines perform sub-optimally in such regions. Measuring plasma HCMV-IgM prior to vaccination provided a clear temporal relationship between HCMV infection and vaccine immunogenicity. However, some limitations exist. The sample size might not have been sufficiently large to detect more subtle differences across the infant groups. Also, the study does not delve into potential biological mechanisms underlying the observed association in HEU infants, which might provide clearer insights. We determined HCMV infection based on HCMV-IgM without confirmation of DNAemia by molecular methods, which could have introduced classification bias. HCMV-IgM serology can identify infant HCMV-specific responses in early life, as opposed to passively acquired transplacental maternal HCMV-IgG antibodies, but may show false positives due to cross reactivity with other herpesviruses such as EBV [37]. HCMV-IgM can also be transient and cleared rapidly in some individuals, thus contributing to misclassification of HCMV status [38]. We also acknowledge that the phenomena of reduced oral rotavirus vaccine immunogenicity may occur in areas with lower HIV seroprevalence and thus limit the generalizability of our findings. Furthermore, genetic, environmental and maternally derived factors known to influence oral rotavirus vaccine immunogenicity [5] were not included in this analysis but which may be involved in the immune responses observed.

Our findings shed light on a potentially critical area of vaccine research, especially in Zambia where HIV prevalence is high. More robustly designed studies may be needed to verify the observed association between HCMV infection and reduced oral rotavirus vaccine response in HEU infants. It would be pertinent to investigate the underlying immunological mechanisms that may be driving this impaired response. Also, it would be useful to study other vaccines’ immunogenicity in relation to HCMV within the HEU infant population to understand if this observation is rotavirus vaccine-specific or a broader immunological phenomenon. If future studies corroborate these findings, it could have profound implications for vaccine policy in regions with high HIV prevalence. There might be a need to revisit vaccine schedules, dosages, or even the development of specific vaccine formulations tailored to the needs of HEU infants. Additionally, understanding such interactions can guide health campaigns and interventions, especially in low-income settings where both HCMV and HIV are prevalent. Public health officials might need to consider additional interventions or strategies to enhance vaccine efficacy in vulnerable subpopulations.

## Conclusion

While the broader implications of HCMV infections on oral rotavirus vaccine response might be limited in the general infant population, the potential impact on HEU infants cannot be overlooked. This study highlights the complexity of immunological responses and the need for targeted interventions to ensure vaccine efficacy, especially in vulnerable subpopulations.

## Supplementary Material

uxae029_suppl_Supplementary_Tables_S1-S2

## Data Availability

The data presented in this study are available on request from the corresponding author. The data are not publicly available due to institutional data policy restrictions.

## Clinical trial registration

The parent trial is registered under the Pan African Clinical Trial Registry as PACTR201804003096919.

## References

[1] Troeger C , Blacker BF, Khalil IA, et al. Estimates of the global, regional, and national morbidity, mortality, and aetiologies of diarrhoea in 195 countries: a systematic analysis for the Global Burden of Disease Study 2016. Lancet Infect Dis 2018, 18, 1211–28. 30243583 10.1016/S1473-3099(18)30362-1 PMC6202444

[2] Bergman H , Henschke N, Hungerford D, et al. Vaccines for preventing rotavirus diarrhoea: vaccines in use. Cochrane Database Syst Rev 2021, 11, CD008521. 34788488 10.1002/14651858.CD008521.pub6 PMC8597890

[3] Burnett E , Parashar UD, Tate JE. Global impact of rotavirus vaccination on diarrhea hospitalizations and deaths among children <5 years old: 2006-2019. J Infect Dis 2020, 222, 1731–9. doi:10.1093/infdis/jiaa081 32095831 PMC7483971

[4] Mwenda JM , Hallowell BD, Parashar U, Shaba K, Biey JN, Weldegebriel GG, et al. Impact of rotavirus vaccine introduction on rotavirus hospitalizations among children under 5 years of age—World Health Organization African Region, 2008–2018. Clin Infect Dis 2021, 73, 1605–8. doi:10.1093/cid/ciab520 34089588 PMC11703079

[5] Lee B. Update on rotavirus vaccine underperformance in low- to middle-income countries and next-generation vaccines. Hum Vaccin Immunother 2021, 17, 1787–802. doi:10.1080/21645515.2020.1844525 33327868 PMC8115752

[6] Mpabalwani EM , Simwaka CJ, Mwenda JM, Mubanga CP, Monze M, Matapo B, et al. Impact of rotavirus vaccination on diarrheal hospitalizations in children aged <5 years in Lusaka, Zambia. Clin Infect Dis 2016, 62, S183–7. doi:10.1093/cid/civ1027 27059354 PMC11977292

[7] Mpabalwani EM , Simwaka JC, Mwenda JM, Matapo B, Parashar UD, Tate JE. Sustained impact of rotavirus vaccine on rotavirus hospitalisations in Lusaka, Zambia, 2009-2016. Vaccine 2018, 36, 7165–9. doi:10.1016/j.vaccine.2018.02.077 29793891 PMC11973831

[8] Chilengi R , Simuyandi M, Beach L, Mwila K, Becker-Dreps S, Emperador DM, et al. Association of maternal immunity with rotavirus vaccine immunogenicity in Zambian infants. PLoS One 2016, 11, e0150100. doi:10.1371/journal.pone.0150100 26974432 PMC4790930

[9] Chilengi R , Mwila- Kazimbaya K, Chirwa M, Sukwa N, Chipeta C, Velu RM, et al. Immunogenicity and safety of two monovalent rotavirus vaccines, ROTAVAC® and ROTAVAC 5D® in Zambian infants. Vaccine 2021, 39, 3633–40. doi:10.1016/j.vaccine.2021.04.060 33992437 PMC8204902

[10] Gatherer D , Depledge DP, Hartley CA, Szpara ML, Vaz PK, Benkő M, et al. ICTV Virus taxonomy profile: Herpesviridae 2021. J Gen Virol 2021, 102, 001673. doi:10.1099/jgv.0.001673 34704922 PMC8604186

[11] Zuhair M , Smit GSA, Wallis G, Jabbar F, Smith C, Devleesschauwer B, et al. Estimation of the worldwide seroprevalence of cytomegalovirus: A systematic review and meta-analysis. Rev Med Virol 2019, 29, e2034. doi:10.1002/rmv.2034 30706584

[12] Bates M , Brantsaeter AB. Human cytomegalovirus (CMV) in Africa: a neglected but important pathogen. J Virus Erad 2016, 2, 136–42. 27482452 10.1016/S2055-6640(20)30456-8 PMC4967964

[13] Gompels UA , Larke N, Sanz-Ramos M, Bates M, Musonda K, Manno D, et al.; CIGNIS Study Group. Human cytomegalovirus infant infection adversely affects growth and development in maternally HIV-exposed and unexposed infants in Zambia. Clin Infect Dis 2012, 54, 434–42. doi:10.1093/cid/cir837 22247303 PMC3258277

[14] Falconer O , Newell ML, Jones CE. The Effect of human immunodeficiency virus and cytomegalovirus infection on infant responses to vaccines: a review. Front Immunol 2018, 9, 328. doi:10.3389/fimmu.2018.00328 29552009 PMC5840164

[15] Cox M , Adetifa JU, Noho-Konteh F, Njie-Jobe J, Sanyang LC, Drammeh A, et al. Limited impact of human cytomegalovirus infection in African Infants on vaccine-specific responses following diphtheria-tetanus-pertussis and measles vaccination. Front Immunol 2020, 11, 1083. 32582177 10.3389/fimmu.2020.01083 PMC7291605

[16] Holder B , Miles DJC, Kaye S, Crozier S, Mohammed NI, Duah NO, et al. Epstein-Barr virus but not cytomegalovirus is associated with reduced vaccine antibody responses in Gambian Infants. PLoS One 2010, 5, e14013. doi:10.1371/journal.pone.0014013 21103338 PMC2984441

[17] Miles DJ , Sanneh M, Holder B, Crozier S, Nyamweya S, Touray ES, et al. Cytomegalovirus infection induces T-cell differentiation without impairing antigen-specific responses in Gambian infants. Immunology 2008, 124, 388–400. doi:10.1111/j.1365-2567.2007.02787.x 18194268 PMC2440833

[18] Pathirana J , Kwatra G, Maposa I, Groome MJ, Madhi SA. Effect of cytomegalovirus infection on humoral immune responses to select vaccines administered during infancy. Vaccine 2021, 39, 4793–9. doi:10.1016/j.vaccine.2021.05.066 34275675

[19] Smith C , Moraka NO, Ibrahim M, Moyo S, Mayondi G, Kammerer B, et al. Human immunodeficiency virus exposure but not early cytomegalovirus infection is associated with increased hospitalization and decreased memory T-cell responses to tetanus vaccine. J Infect Dis 2020, 221, 1167–75. doi:10.1093/infdis/jiz590 31711179 PMC7075416

[20] Sanz-Ramos M , Manno D, Kapambwe M, Ndumba I, Musonda KG, Bates M, et al.; CIGNIS study team. Reduced Poliovirus vaccine neutralising-antibody titres in infants with maternal HIV-exposure. Vaccine 2013, 31, 2042–9. doi:10.1016/j.vaccine.2013.02.044 23474309

[21] Laban NM , Bosomprah S, Simuyandi M, Chibuye M, Chauwa A, Chirwa-Chobe M, et al. Evaluation of ROTARIX&reg; Booster dose vaccination at 9 months for safety and enhanced anti-rotavirus immunity in Zambian children: A Randomised Controlled Trial. Vaccines 2023, 11, 346. doi:10.3390/vaccines11020346 36851224 PMC9960729

[22] Angel J , Steele AD, Franco MA. Correlates of protection for rotavirus vaccines: possible alternative trial endpoints, opportunities, and challenges. Hum Vaccin Immunother 2014, 10, 3659–71. doi:10.4161/hv.34361 25483685 PMC4514048

[23] Miles DJC , Sande M, Jeffries D, et al. Cytomegalovirus infection in Gambian infants leads to profound CD8 T-cell differentiation. J Virol 2007, 81, 5766–76. 17376923 10.1128/JVI.00052-07 PMC1900274

[24] Goodier MR , Jonjić S, Riley EM, Juranić Lisnić V. CMV and natural killer cells: shaping the response to vaccination. Eur J Immunol 2018, 48, 50–65. doi:10.1002/eji.201646762 28960320

[25] Tuengel J , Ranchal S, Maslova A, Aulakh G, Papadopoulou M, Drissler S, et al. Characterization of adaptive-like γδ T cells in Ugandan infants during primary cytomegalovirus infection. Viruses 2021, 13, 1987. doi:10.3390/v13101987 34696417 PMC8537190

[26] Crough T , Khanna R. Immunobiology of human cytomegalovirus: from bench to bedside. Clin Microbiol Rev 2009, 22, 76–98, Table of Contents. doi:10.1128/CMR.00034-08 19136435 PMC2620639

[27] Kalejta RF. Tegument proteins of human cytomegalovirus. Microbiol Mol Biol Rev 2008, 72, 249–65, table of contents. doi:10.1128/MMBR.00040-07 18535146 PMC2415745

[28] Patro ARK. Subversion of immune response by human cytomegalovirus. Front Immunol 2019, 10, 1155. doi:10.3389/fimmu.2019.01155 31244824 PMC6575140

[29] Jackson SE , Mason GM, Wills MR. Human cytomegalovirus immunity and immune evasion. Virus Res 2011, 157, 151–60. doi:10.1016/j.virusres.2010.10.031 21056604

[30] Afran L , Garcia Knight M, Nduati E, Urban BC, Heyderman RS, Rowland-Jones SL. HIV-exposed uninfected children: a growing population with a vulnerable immune system? Clin Exp Immunol 2014, 176, 11–22. doi:10.1111/cei.12251 24325737 PMC3958150

[31] Filteau S , Rowland-Jones S. Cytomegalovirus infection may contribute to the reduced immune function, growth, development, and health of HIV-exposed, uninfected African children. Front Immunol 2016, 7, 257. doi:10.3389/fimmu.2016.00257 27446087 PMC4928134

[32] Sbihi H , Simmons KE, Sears MR, Moraes TJ, Becker AB, Mandhane PJ, et al. Early-life cytomegalovirus infection is associated with gut microbiota perturbations and increased risk of atopy. Pediatr Allergy Immunol 2022, 33, e13658. doi:10.1111/pai.13658 34467574

[33] Le-Trilling VTK , Ebel JF, Baier F, Wohlgemuth K, Pfeifer KR, Mookhoek A, et al. Acute cytomegalovirus infection modulates the intestinal microbiota and targets intestinal epithelial cells. Eur J Immunol 2023, 53, e2249940. doi:10.1002/eji.202249940 36250419

[34] Harris VC , Armah G, Fuentes S, Korpela KE, Parashar U, Victor JC, et al. Significant correlation between the infant gut microbiome and rotavirus vaccine response in rural Ghana. J Infect Dis 2016, 215, 34–41. doi:10.1093/infdis/jiw518 27803175 PMC5225256

[35] Machiavelli A , Duarte RTD, Pires MMS, Zárate-Bladés CR, Pinto AR. The impact of in utero HIV exposure on gut microbiota, inflammation, and microbial translocation. Gut Microbes 2019, 10, 599–614. doi:10.1080/19490976.2018.1560768 30657007 PMC6748604

[36] Kampmann B , Jones CE. Factors influencing innate immunity and vaccine responses in infancy. Philos Trans R Soc Lond B Biol Sci 2015, 370, 20140148. doi:10.1098/rstb.2014.0148 25964459 PMC4527392

[37] Lang D , Vornhagen R, Rothe M, Hinderer W, Sonneborn HH, Plachter B. Cross-Reactivity of Epstein-Barr virus-specific immunoglobulin M antibodies with cytomegalovirus antigens containing glycine homopolymers. Clin Diagn Lab Immunol 2001, 8, 747–56. doi:10.1128/CDLI.8.4.747-756.2001 11427421 PMC96137

[38] Sarasini A , Arossa A, Zavattoni M, Fornara C, Lilleri D, Spinillo A, et al. Pitfalls in the serological diagnosis of primary human cytomegalovirus infection in pregnancy due to different kinetics of IgM clearance and IgG avidity index maturation. Diagnostics (Basel) 2021, 11, 396. doi:10.3390/diagnostics11030396 33652709 PMC7996894

