# Supplementary material for: Human cytomegalovirus seropositivity and its influence on oral rotavirus vaccine immunogenicity: a specific concern for HIV-exposed-uninfected infants
Source: Clin Exp Immunol. 2024 Mar 28;217(1):99–108. doi: 10.1093/cei/uxae029 (PMC11188542; doi:10.1093/cei/uxae029)
Supplement: uxae029_suppl_Supplementary_Tables_S1-S2 [file uxae029_suppl_supplementary_tables_s1-s2.docx]

**Supplementary Table 1. Geometric mean RV-IgA titre at 12 months by baseline characteristics of infants**

|  | **Number of infants,** | **RV-IgA (units/mL) at 12 months** |  |
| --- | --- | --- | --- |
| **Characteristics** | n (% of total) | GMT (95% CI) | p-value |
| Sex |  |  |  |
| Female | 73 (47.1) | 19.9 (11.3, 35.0) | 0.312 |
| Male | 82 (52.9) | 30.1 (17.0, 53.3) |  |
| Gestation |  |  |  |
| Full-term | 146 (94.2) | 26.1 (17.3, 39.3) | 0.290 |
| Pre-term | 9 (5.8) | 10.43 (1.3, 85.5) |  |
| Mode of Delivery |  |  |  |
| Caesarean | 8 (5.2) | 13.3 (2.1, 83.1) | 0.473 |
| Vaginal | 147 (94.8) | 25.6 (16.9, 38.7) |  |
| Feeding |  |  |  |
| Breastmilk | 145 (93.5) | 25.2 (16.6, 38.3) | 0.724 |
| Breastmilk + formula | 10 (6.5) | 18.8 (3.7, 95.5) |  |
| Birth weight, kg (n=154) |  |  |  |
| <2.5 | 16 (10.4) | 44.3 (10.9, 180.0) | 0.323 |
| ≥2.5 | 138 (89.6) | 22.9 (15.0, 34.9) |  |
| Stunting (LAZ <-2) |  |  |  |
| No | 129 (83.2) | 23.2 (15.1, 35.5) | 0.469 |
| Yes | 26 (16.8) | 34.4 (10.8, 109.2) |  |
| Wasting (WLZ <-2) |  |  |  |
| No | 152 (98.1) | 25.6 (17.1, 38.3) | 0.227 |
| Yes | 3 (1.9) | 4.3 (0.01, 2391.3) |  |
| Maternal HIV (n=154) |  |  |  |
| negative | 107 (69.5) | 20.6 (13.0, 32.5) | 0.148 |
| positive | 47 (30.5) | 39.0 (17.2, 88.5) |  |
| Toilet facility sharing across households |  |  |  |
| not shared | 32 (20.7) | 59.4 (23.2, 151.9) | 0.027 |
| shared | 123 (79.3) | 19.7 (12.7, 30.6) |  |
| Water source |  |  |  |
| piped into household | 55 (35.5) | 28.7 (15.1, 54.5) | 0.592 |
| wells/public taps and boreholes | 100 (64.5) | 22.8 (13.6, 38.3) |  |
| Number of children in household |  |  |  |
| 1-3 | 122 (78.7) | 22.7 (14.5, 35.4) | 0.665 |
| 4-6 | 29 (18.7) | 32.6 (11.6, 91.7) |  |
| 7-9 | 4 (2.6) | 50.7 (1.9, 1334.1) |  |
| **Total** | **155 (100)** | **24.76 (16.60, 36.92)** |  |

**Supplementary Table 2. Percent of infant with four-fold increase in RV-IgA between 9 and 12 months by baseline characteristics.**

|  | **Number of infants,** | **Four-fold increase in RV-IgA between 9 and 12 months** |  |
| --- | --- | --- | --- |
| **Characteristics** | n (% of total) | n (%) | p-value |
| Sex |  |  |  |
| Female | 73 (47.1) | 31 (50.8) | 0.511 |
| Male | 82 (52.9) | 30 (49.2) |  |
| Gestation |  |  |  |
| Full-term | 146 (94.2) | 58 (95.1) | 1.000 |
| Pre-term | 9 (5.8) | 3 (4.9) |  |
| Mode of Delivery |  |  |  |
| Caesarean | 8 (5.2) | 3 (4.9) | 1.000 |
| Vaginal | 147 (94.8) | 58 (95.1) |  |
| Feeding |  |  |  |
| Breastmilk | 145 (93.5) | 57 (93.4) | 1.000 |
| Breastmilk + formula | 10 (6.5) | 4 (6.6) |  |
| Birth weight, kg (n=154) |  |  |  |
| <2.5 | 6 (10.4) | 6 (9.8) | 1.000 |
| ≥2.5 | 138 (89.6) | 55 (90.2) |  |
| Stunting (LAZ <-2) |  |  |  |
| No | 129 (83.2) | 49 (80.3) | 0.511 |
| Yes | 26 (16.8) | 12 (19.7) |  |
| Wasting (WLZ <-2) |  |  |  |
| No | 152 (98.1) | 61 (100.0) | 0.279 |
| Yes | 3 (1.9) | 0 (0.0) |  |
| Maternal HIV (n=154) |  | n=60 |  |
| negative | 107 (69.5) | 41 (68.3) | 0.858 |
| positive | 47 (30.5) | 19 (31.7) |  |
| Toilet facility sharing across households |  |  |  |
| not shared | 32 (20.7) | 16 (26.2) | 0.223 |
| shared | 123 (79.3) | 45 (73.8) |  |
| Water source |  |  |  |
| piped into household | 55 (35.5) | 22 (36.1) | 1 |
| wells/public taps and boreholes | 100 (64.5) | 39 (63.9) |  |
| Number of children in household |  |  |  |
| 1-3 | 122 (78.7) | 43 (70.5) | 0.825 |
| 4-6 | 29 (18.7) | 15 (24.6) |  |
| 7-9 | 4 (2.6) | 3 (4.9) |  |
| **Total** | **155 (100)** | **61 (100))** |  |
